# Ultrawide Color Gamut Perovskite and CdSe/ZnS Quantum-Dots-Based White Light-Emitting Diode with High Luminous Efficiency

**DOI:** 10.3390/nano9091314

**Published:** 2019-09-14

**Authors:** Chih-Hao Lin, Chieh-Yu Kang, Akta Verma, Tingzhu Wu, Yung-Min Pai, Tzu-Yu Chen, Chun-Lin Tsai, Ya-Zhu Yang, S.K. Sharma, Chin-Wei Sher, Zhong Chen, Po-Tseng Lee, Shu-Ru Chung, Hao-Chung Kuo

**Affiliations:** 1 Department of Photonics and Institute of Electro-Optical Engineering, College of Electrical and Computer Engineering, National Chiao Tung University, Hsinchu 30010, Taiwan; 2 Department of Applied Physics, Indian Institute of Technology (Indian School of Mines), Dhanbad-826004, India; 3 Department of Electronic Science, Fujian Engineering Research Center for Solid-State Lighting, Xiamen University, Xiamen 361005, China; 4 Department of Materials Science and Engineering, National Formosa University, Yunlin 63201, Taiwan

**Keywords:** ultrawide color gamut display, perovskite quantum dots, white LED

## Abstract

We demonstrate excellent color quality of liquid-type white light-emitting diodes (WLEDs) using a combination of green light-emitting CsPbBr_3_ and red light-emitting CdSe/ZnS quantum dots (QDs). Previously, we reported red (CsPbBr_1.2_I_1.8_) and green (CsPbBr_3_) perovskite QDs (PQDs)-based WLEDs with high color gamut, which manifested fast anion exchange and stability issues. Herein, the replacement of red PQDs with CdSe/ZnS QDs has resolved the aforementioned problems effectively and improved both stability and efficiency. Further, the proposed liquid-type device possesses outstanding color gamut performance (132% of National Television System Committee and 99% of Rec. 2020). It also shows a high efficiency of 66 lm/W and an excellent long-term operation stability for over 1000 h.

## 1. Introduction

White light-emitting diodes (WLEDs), also known as solid-state devices, have recently been intensively investigated. The major advantages of WLEDs are low-power consumption and long lifetime resulting in their great potential for lighting, signal, and display applications [1,2,3,4,5]. Many important phosphors played key roles in color conversion function to fulfill several applications in the last two decades [6,7,8,9]. Recently, colloidal quantum-dot–light-emitting-diodes (QD-LEDs) have been rapidly developed for display and lighting applications because of their narrow bandwidths, high luminescence efficiency, broad absorption, and size-engineered bandgap [10,11,12,13,14,15,16,17]. A promising class of such materials is metal-halide perovskites [18,19,20]. The CsPbX_3_ (X = Cl, Br, or I) perovskite was reported by Moller in 1958 [21]. The success of perovskites in light-emitting devices depends on finding reliable strategies to tune the bandgap. Thus far, color can be tuned chemically by applying mixed halide stoichiometry [19,22]. In our previous work, we used green (CsPbBr_3_) and red (CsPbBr_1.2_I_1.8_) PQDs to fabricate WLED devices and the result showed that the excellent device performance can be achieved with a wide color gamut. Despite owing wide color gamut, we suffered with low luminous efficiency and poor stability of the proposed device [23]. These problems led us to modify the device structure. Consequently, in this present study, we have replaced CsPbBr_1.2_I_1.8_ red PQDs with CdSe/ZnS QDs because of the high quantum yield (QY) and narrow full width at half maxima (FWHM) of CdSe/ZnS QDs. Furthermore, the CdSe/ZnS core-shell QDs have high fluorescent efficiency [24,25,26,27]. The ZnS shell plays a crucial role in emission properties and enhances the chemical stability and photostability of QDs [28,29]. The core is the emission layer and the function of the shell is to limit and reduce surface defects and confine electron–hole pairs in the core-shell structure. Typically, the particle size of CdSe QDs ranges from 2 nm to 10 nm, resulting in the coverage of luminescence wavelength from visible light to near-infrared light [30]. The color of light can be controlled by altering the particle size meaning that the small CdSe quantum dots produce a shorter wavelength of light [31].

In the present work, two types of WLED device structures are fabricated for comparison: hybrid-type (red CdSe/ZnS QDs solid film and green CsPbBr_3_ PQDs liquid) and liquid-type (mixing of green PQDs and red CdSe/ZnS QDs). Both prepared devices are excited using standard blue LEDs. The experiment results demonstrate that the liquid-type WLED achieves an outstanding color gamut performance with National Television System Committee (NTSC) higher than 132% and ITU-R Recommendation BT.2020 (Rec. 2020) of 99%. In addition, the luminous efficiency of the liquid-type WLED is 29% higher than that of the hybrid-type sample, and the attenuation is only 4% after 1000 h of operation.

## 2. Materials and Methods

### 2.1. Characterization

The optical properties of QDs were measured using an ultraviolet-visible spectrometer (UV-Vis, Hitachi UH5300 from Saitama, Kami, Japan) and a fluorescence spectrophotometer (FL, Hitachi F-7000, Saitama, Kami, Japan). The integrating sphere, spectrometer and power supplies (Agilent E3632A, Keithley 2400, Motech DR2004, Santa Clara, CA, USA) were used to measure optical and electrical characteristics of two types of WLEDs devices. To evaluate the QY of green PQDs, the concentrations of PQDs and Rhodamine 6G (R6G) dye were adjusted to the same optical density at the same excitation wavelength. Relative QYs were obtained by comparing to a standard R6G (QY 95%) in methanol. The QY of samples was calculated using the following equation [32]:

(1) $$QY_{s}=\frac{A_{r}F_{r}n_{r}{}^{2}}{A_{s}F_{s}n_{s}{}^{2}}QY_{r}$$

Here, *F_s_* and *F_r_* are the integrated fluorescence emission areas of the sample and reference. *A_s_* and *A_r_* are the absorbances at the same excitation wavelength of the sample and reference, *n_s_* and *n_r_* denote the solvent refractive indices of the sample and reference, and *QY_s_* and *QY_r_* denote the QY of the sample and reference, respectively. The QY of CdSe/ZnS is greater than 50% as proposed by the UT dots Corporation. The FWHM of used green PQDs and red CdSe/ZnS QDs are 19 nm and 25 nm, respectively.

### 2.2. Materials and Device Fabrication

**Figure 1:**
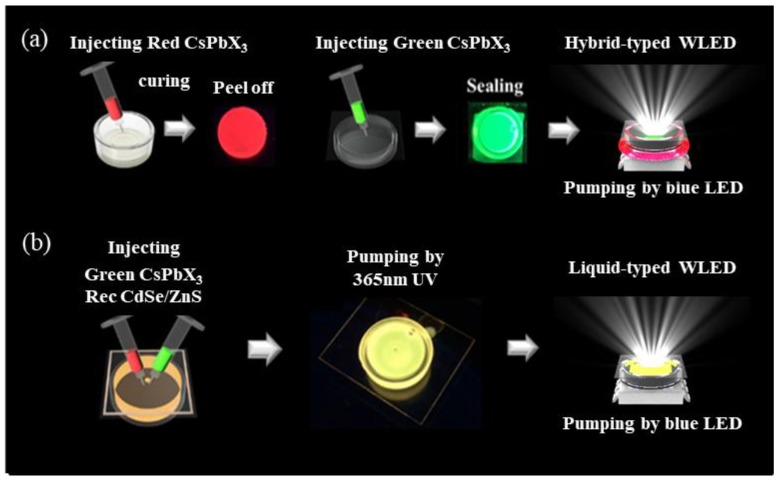
Process flowchart of (**a**) hybrid-type and (**b**) liquid-type QD-based WLEDs.

The inorganic green PQDs used in this study were synthesized using the hot-injection methods. A few grams of PbBr_2_ and 10 mL of octadecene (ODE) were loaded into a three-necked flash and vacuum dried at 120 °C for 1 h. Next, oleylamine (OLA) and oleic acid (OA) were injected into the flask under Ar atmosphere and mixed until completely dissolved. Further, we raised the reaction temperature to 180 °C and quickly injected the prepared Cs-oleate solution into the flask. After 5 s of reaction, the obtained reaction mixture was cooled down to 27 °C using an ice-water bath. Finally, the green PQDs were centrifuged, the supernatant was abandoned, and the precipitant was re-dissolved into the solvent. The CdSe/ZnS QDs were purchased from the UT-dots Corporation. Now, for the fabrication of a hybrid-type device as shown in Figure 1, a lead frame-type blue LED of width 5.0 mm and length 7.0 mm was filled with poly (dimethyloxane) (PDMS) using the dispensing machine, and baked at 70 °C for 2 h. The pumping source, blue LED, has an emission wavelength of 450 nm with a chip size of 45 mil × 45 mil. The film is produced as follows: the CdSe/ZnS QDs were dissolved in toluene to an appropriate concentration and then mixed with PDMS in a glass box of inner radius 9 mm and outer radius 10 mm. Next, they were vacuumed and baked at 30 °C for a few hours. The fully dried films were peeled off from the box. Further, for the packaging of the liquid-type green CsPbBr_3_, a glass box of inner radius 6 mm and outer radius 7 mm was used. A hole was drilled in the middle of the thin glass using a laser to reduce the gap in the device. Next, the thin glass was pasted on the glass box. Finally, the QD solution was injected into the glass box and the compartment was sealed to produce the liquid device. The film and the liquid device were combined to form hybrid WLEDs. Moreover, in order to prepare the liquid-type WLEDs, a glass box of radius approximately 7 mm was used. A laser was used to drill a hole in the middle of the thin glass to facilitate the injection of the QD solution and reduce the device gap. A thin glass was then attached to the glass box, the QD solution was injected, and the compartment was sealed to fabricate a liquid-type device.

## 3. Results and Discussion

**Figure 2:**
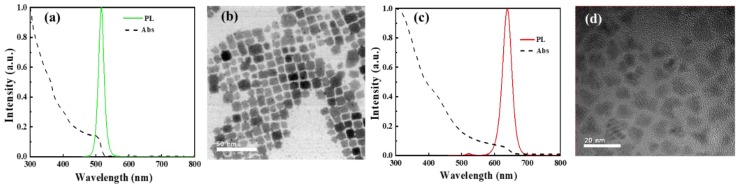
(**a**) UV-Vis absorption, PL emission spectrum, and (**b**) TEM images of CsPbBr3 green perovskite quantum dots (QDs). (**c**) UV-Vis absorption, PL emission spectrum, and (**d**) TEM images of red CdSe/ZnS QDs.

Figure 2 represents the photoluminescence (PL) emission spectrum, UV-visible absorption spectrum, and TEM images of green PQDs and red CdSe/ZnS QDs. The PL emission spectrum of green PQDs (Figure 2a) and CdSe/ZnS QDs (Figure 2c) are recorded at excitation wavelengths of 370 nm and 365 nm, respectively. In the case of green PQDs, the sharp absorption edge and PL emission peak are located at 505 nm and 520 nm, respectively. In the case of red CdSe/ZnS QDs, a broad absorption band is observed near 629 nm and a sharp PL emission peak is centered at 640 nm [33,34]. Further, the particle size of both red and green PQDs (Figure 2b) and red CdSe/ZnS QDs (Figure 2d) are found to be 9.7 and 6.7 nm, respectively, using TEM images.

**Figure 3:**
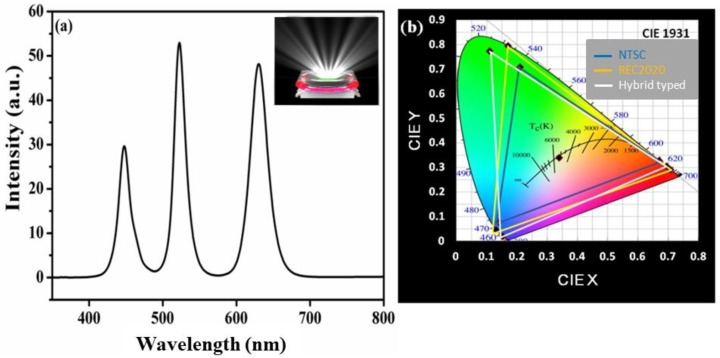
(**a**) Electroluminescence (EL) spectrum of hybrid-type WLED and (**b**) color coordinates and color gamut of cold white LED on the CIE1931 chromaticity diagram.

**Figure 4:**
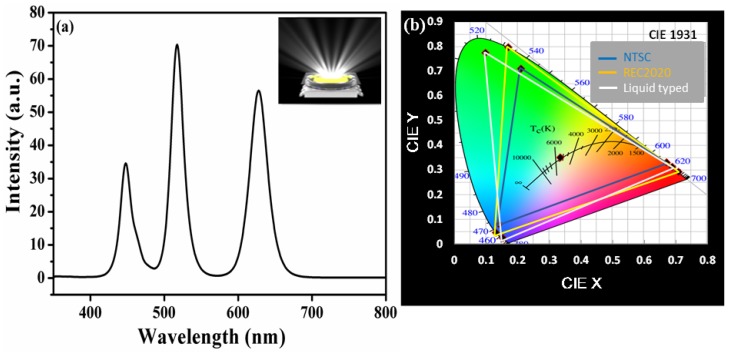
(**a**) EL spectrum of liquid-type WLED and (**b**) color coordinates and color gamut of cold white LED on the CIE1931 chromaticity diagram.

Figure 3 and Figure 4 represent the electroluminescence (EL) spectrum and CIE color gamut compared to the NTSC and Rec. 2020 standards for both prepared hybrid-type and liquid-type device structures. The total emission spectrum generated by WLEDs consists of three peaks located at 450 nm, 522 nm, and 630 nm corresponding to blue LED chip, green PQDs, and red CdSe/ZnS QDs, respectively. Furthermore, it was found that hybrid-type WLEDs consisting of CdSe/ZnS red QD (film) and combined with green PQDs (liquid) showed a luminous efficiency of 51 lm/W and CIE coordinates of (0.33, 0.34) at driving current of 10 mA. The obtained value of NTSC is 122% of standard and Rec. 2020 of 91% [35]. A cold WLED with correlated color temperature (CCT) of 5205 K is finally achieved, as shown in the inset of Figure 3a. It is worth to mention here that the replacement of red PQDs with CdSe/ZnS QDs not only provides a larger value of color gamut but also resolves the anion-exchange issue that previously occurred in case of both red and green PQDs liquid-type device structures. Hence, we further put efforts to improve the luminous efficiency and stability through a liquid-type device structure under the premises of a wide color gamut. In order to fabricate a liquid-type device structure, we mixed CdSe/ZnS red QDs and green PQDs in a sealed glass box (manufacturing process is described in the device fabrication section). The liquid-type structure is also pumped using a blue LED to produce white light. The EL spectrum and CIE coordinates of the liquid-type WLED are shown in Figure 4a,b, respectively. The result shows that the CIE and CCT of the device are (0.33, 0.35) and 5445 K. The luminous efficiency of the liquid-type device is 66 lm/W at the driving current of 10 mA, which is 29% higher than that of the hybrid-type device structure, which is 51 lm/W.

Recently, Yang and Chung presented a high color quality (132% of NTSC) WLED device based on green and red PQD films and indicated a promised potential by applying PQDs for QDs display [36]. Sadeghi et al. reported a high efficiency liquid-type WLED device with an efficiency of 64 lm/W and a stable life test of 100 h. However, its color gamut was narrower due to insufficient vividness of green emission of 550 nm [37]. Table 1 shows the comparison result of hybrid-type and liquid-type WLEDs used in this study. The liquid-type WLED shows a better performance not only for color purity (132% of NTSC and 99% of Rec. 2020), which is 8.5% and 9.6% greater than the value of the reference (PQD hybrid-type WLEDs) samples but it also shows a high luminous efficiency of 66 lm/W. It shows a great improvement in 1000 h stability with higher color quality and efficiency compared to the previous best works. For liquid-type WLEDs, we have replaced CsPbBr_1.2_I_1.8_ red PQDs with CdSe/ZnS QDs because of the higher QY (50%) and narrow FWHM of CdSe/ZnS QDs (25 nm). For PQD hybrid-type WLEDs, red-emitting PQDs only have 30% QY and 33 nm FWHM [21]. These two characteristics improve the luminous efficiency and color performance of liquid-type WLEDs. Furthermore, the ZnS shell of red-emitting QD not only plays a crucial role in the emission properties but also enhances the chemical stability and photostability of QDs [28,29]. It limits and reduces surface defects and confines electron–hole pairs in the core-shell structure and there is no anion exchange effect in the device with the combination of green light-emitting CsPbBr_3_ and red light-emitting CdSe/ZnS QDs. Therefore, the liquid-type WLEDs have a better light stability in 1000 h compared to the PQD hybrid-type WLEDs.

**Figure 5:**
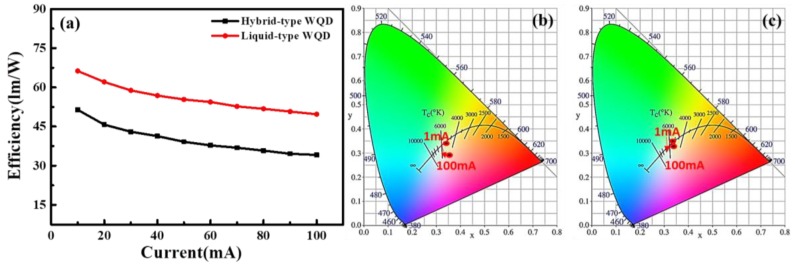
(**a**) Efficiency of two fabricated structures at varying currents. (**b**) Hybrid-type and (**c**) liquid-type WLEDs in CIE 1931 chromaticity coordinates at varying currents.

Figure 5 represents the current-dependent efficiencies comparison between two fabricated hybrid-type and liquid-type devices. It can be clearly seen from Figure 5a that the liquid-type device has higher luminous efficiency than the hybrid-type device structure. Further, it can also be noticed that by varying currents, the efficiency of the liquid-type device decays by 25%, whereas that of the hybrid-type decays by 34%. Figure 5b,c represent the CIE 1931 chromaticity coordinates comparison between the hybrid-type and liquid-type devices, respectively, at the current ranging from 10 mA to 100 mA. It can be clearly seen that the color coordinates of the liquid-type WLED are offset much less than the hybrid-typed device. On the basis of the above results, it can be concluded that the liquid-type WLED is suitable for high-current operation than the hybrid-type. Consequently, the liquid-type WLED can maintain good efficiency and light quality. Furthermore, the hybrid-type device structure has a more pronounced layered illumination, whereas the liquid-type device has better illumination uniformity that allows the device to be used on the backend application [38].

**Figure 6:**
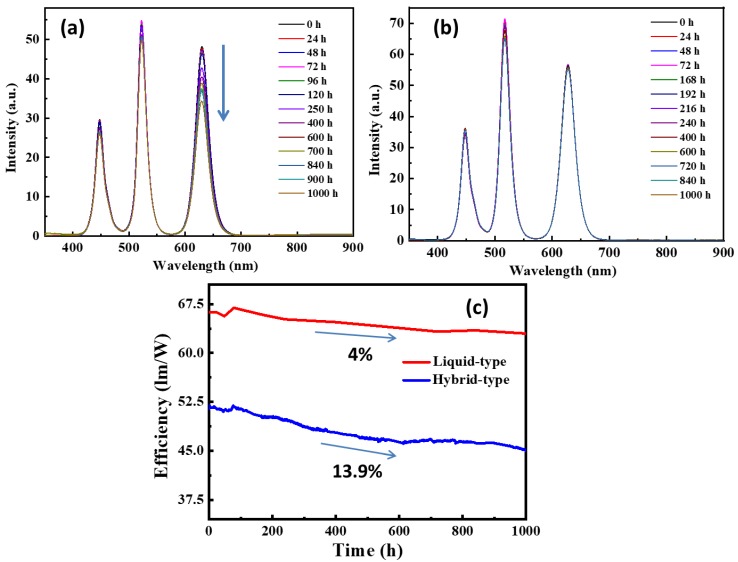
The emission spectra of (**a**) hybrid-type WLED and (**b**) liquid-type WLED under various operation times; (**c**) luminous efficiency versus the operation time from 0 h to 1000 h.

Figure 6a,b show the emission spectra of the hybrid-type and liquid-type WLEDs device structures for various time operations at room temperature. After performing the initial characterizations, it is important to test the longevity of both fabricated devices. In the present case, the measurement time was set from 0 h to 1000 h. Figure 6c shows the efficiency decay of the hybrid-type WLED structure and liquid-type WLED structure in the time period ranging from 0 h to 1000 h. Specifically, we made a progress on package sealing compared to previous work, which resulted in better life of the hybrid-type device. It can be depicted from the experiment results that the efficiency reductions of the hybrid-type and liquid-type device structures are 13.9% and 4% after 1000 h of testing, respectively. For PQD hybrid-type WLEDs, it can be observed from Figure 6a that the decay mostly comes from red-emitting PQDs. To improve the stability, we have replaced CsPbBr_1.2_I_1.8_ red PQDs with CdSe/ZnS QDs, which has ZnS shell enhancing the chemical stability and photostability of QDs. Subsequently, we can conclude that the liquid-type WLED has a stable and sustainable performance over a long period of time. Further, the liquid-type packaging not only maintains its intensity but also the same light quality even after 1000 h [39,40].

**Figure 7:**
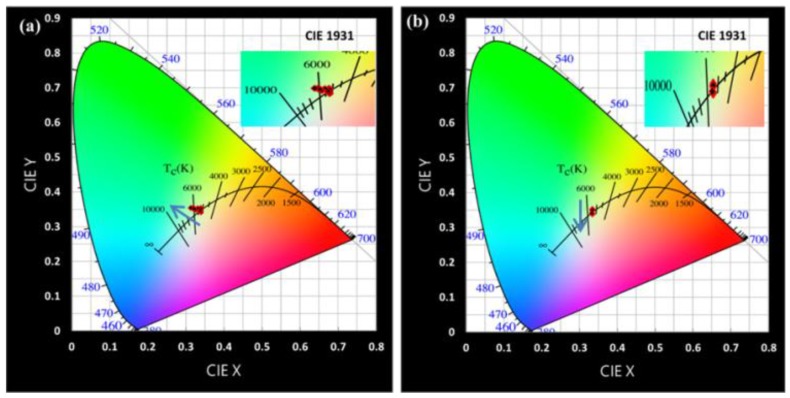
Chromaticity coordinates shift of (**a**) hybrid-type WLED and (**b**) liquid-type WLED from 0 to 1000 h on CIE1931.

**Figure 8:**
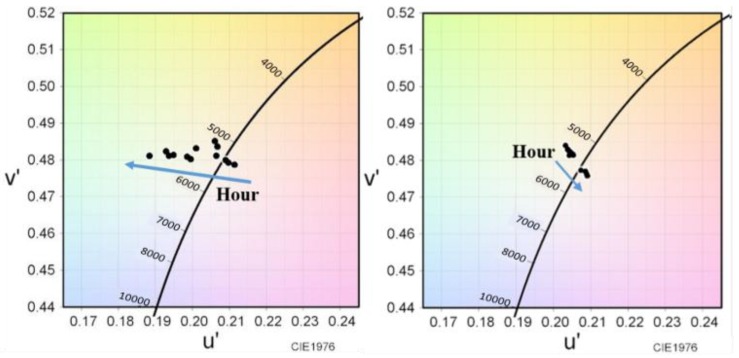
Chromaticity coordinates shift of (**a**) hybrid-type WLED and (**b**) liquid-type WLED from 0 h to 1000 h on CIE1976.

Figure 7 represents the chromaticity coordinate shift performances of the hybrid-type and liquid-type device structures for the 1000 h test. For the QDs-based WLEDs, the color deviation is used to evaluate the color stability for high-quality lighting applications. It can be seen from Figure 8a that the CIE 1931 chromaticity coordinates shift from (0.339, 0.341) to (0.312, 0.354) in case of the hybrid-type WLED. The chromaticity coordinates of the liquid-type WLED shift from (0.334, 0.350) to (0.334, 0.340) under 0 h to 1000 h test, as shown in Figure 8b. Hence, it can be concluded that the chromaticity coordinate offsets of both structures are small, particularly of the liquid-type device. The reason for the shift of the hybrid-type WLED towards blue and green directions is because of the film form of red CdSe/ZnS that causes larger intensity decay. Consequently, it is again confirmed that the liquid form of QDs is more stable than the film form under a long storage time test. The (Δ*u*′*v*′) value is another common approach used to indicate spatial color uniformity. As the color deviation of a lighting system, Δ*u*′*v*′ is calculated as follows:

(2) $$\begin{array}{l} u^{\prime }=4x/(-2x+12y+3)\\ v^{\prime }=9y/(-2x+12y+3)\\ \Delta u^{\prime }v^{\prime }=\sqrt{{\left(\Delta u^{\prime }\right)}^{2}+{\left(\Delta v^{\prime }\right)}^{2}} \end{array}$$

where *u*′ and *v*′ are chromaticity coordinates in the CIE 1976 diagram, and *x* and *y* are chromaticity coordinates in the CIE 1931 diagram. The CIE 1976 chromaticity coordinate shift of both hybrid-type and liquid-type WLEDs from 0 h to 1000 h are shown in Figure 8a,b, respectively. Typically, for display applications, the tolerance for color deviation is approximately 0.01. It can be depicted from these experimental results that the calculated color deviation value of the liquid-type WLED is 0.0068 that is less than the tolerance deviation value [15,41]. Hence, this clearly shows that the liquid-type packaging is much more suitable for the display backlight application.

## 4. Conclusions

In summary, we have fabricated a liquid-type device structure using green PQD and red CdSe/ZnS QDs. Compared to our previous results, the replacement of red PQDs with CdSe/ZnS QDs successfully resolves the anion exchange issue and also provides an outstanding color gamut value with improvement in luminous efficiency. The experiment results depict that the liquid-type WLED device can achieve an outstanding color gamut value (132% of NTSC and 99% of Rec. 2020). Furthermore, the liquid-type structure possesses a luminous efficiency of 29% higher than that of the hybrid-type device structure with only 4% attenuation after 1000 h of storage, showing its great optical property and potential application towards displays and lightings.

## Figures and Tables

**Table 1:** The performance comparison: red film PQD + green liquid PQD WLED (hybrid-type) vs. red CdSe/ZnS liquid QD + green PQD type WLED (liquid-type).

Device Properties	Hybrid-Type WLED	Liquid-Type WLED
NTSC (%)	122	132
Rec. 2020 (%)	91	99
Luminous efficiency (lm/W)	51	66
